# Unexpected benefits of deciding by mind wandering

**DOI:** 10.3389/fpsyg.2013.00598

**Published:** 2013-09-06

**Authors:** Colleen E. Giblin, Carey K. Morewedge, Michael I. Norton

**Affiliations:** ^1^Marketing, Tepper School of Business, Carnegie Mellon UniversityPittsburgh, PA, USA; ^2^Marketing, Harvard Business SchoolBoston, MA, USA

**Keywords:** decision strategies, post-choice satisfaction, mind wandering, spontaneous thought, deliberation

## Abstract

The mind wanders, even when people are attempting to make complex decisions. We suggest that mind wandering—allowing one's thoughts to wander until the “correct” choice comes to mind—can positively impact people's feelings about their decisions. We compare post-choice satisfaction from choices made by mind wandering to reason-based choices and randomly assigned outcomes. Participants chose a poster by mind wandering or deliberating, or were randomly assigned a poster. Whereas forecasters predicted that participants who chose by mind wandering would evaluate their outcome as inferior to participants who deliberated (Experiment 1), participants who used mind wandering as a decision strategy evaluated their choice just as positively as did participants who used deliberation (Experiment 2). In some cases, it appears that people can spare themselves the effort of deliberation and instead “decide by wind wandering,” yet experience no decrease in satisfaction.

The mind regularly wanders while people perform tasks that, at face value, would seem to demand their full attention. Most drivers have had the experience of realizing that they have just been driving on autopilot—that there is a substantial gap in their memory of the drive itself. Mind wandering is not unique to driving (Smallwood and Schooler, 2006; Mason et al., 2007; Killingsworth and Gilbert, 2010). Consider a routine trip to the grocery store. While navigating store aisles, people may occasionally find that products have appeared in their cart while their mind was focused on something else. The mind can be on a similar sort of autopilot while engaging in other spontaneous decision processes. Rather than considering their options carefully, for example, people may choose to eat at the first restaurant that pops into their mind. We propose that people may underestimate satisfaction with decisions made by mind wandering. We show that people anticipate the results of such decision processes to be less satisfying than the results of more deliberate decision processes, but post-choice satisfaction is often as high whether deciding by mind wandering or deliberating.

Most theories of legal and economic decision making suggest that controlled thought processes such as logic, deliberation, and planning are likely to best reflect the intentions, desires, and preferences of the person who is the decision maker (for a review, see Morewedge et al., 2010). Having consciously deliberated choice alternatives and their consequences before making a decision (premeditation) is considered the best indicator of rational action by the legal system and society at large (Denno, 2003). Formal economic theories of decision making such as the rational agent model posit that choosing the best outcome requires that one consider the utility of each alternative and choose the alternative with the highest expected value (Von Neumann and Morgenstern, 1944). These theories acknowledge that in some cases people satisfice—choose an option that meets their threshold of an acceptable outcome. Satisficing is an inferior strategy implemented when one does not have the time or resources to engage a utility maximization strategy: unless by chance, it does not result in choosing the optimal outcome (Simon, 1957; Schwartz et al., 2002). Because decision making by deliberation is thought to best reflect the intentions and maximize the utility of the decision maker, legal and economic theories suggest that people should believe they will be most satisfied with the outcomes of decisions made with deliberate or controlled thinking (Morewedge et al., 2010).

Decisions made by mind wandering do not fall into the category of decisions made through deliberate or controlled thinking. Mind wandering entails, “the conscious processing of information that is unrelated to immediate sensory input and to the task currently being performed” (Smallwood et al., 2011). By definition, decisions made by mind wandering entail a process that does not involve deliberation or controlled thinking about choice alternatives. Instead, decisions made by mind wandering belong to the category of decisions made by spontaneous thought. Spontaneous thought is stimulus independent thought, “streams of thoughts and images unrelated to immediate sensory input” (Teasdale et al., 1995). Other members of this category include random thoughts, dreams, intuition, intrusive thoughts, and Freudian slips (Morewedge et al., in preparation). As mind wandering and most forms of spontaneous thoughts do not typically adhere to the kind of controlled stimulus-dependent thinking that characterizes rational deliberation, we suggest that people will anticipate the outcome of decisions made by mind wandering to be less satisfactory than the outcomes of decisions made by deliberation.

There is reason, however, to suggest that in some cases spontaneous thought processes such as mind wandering might result in outcomes that provide equal or greater satisfaction as outcomes resulting from deliberative thought processes. Previous research has found that various forms of spontaneous thought are believed to be more revealing, providing better access into the mind of the thinker, than similar forms of deliberate thinking. Information revealed in a dream is believed to reveal more meaningful information about the self than the same information revealed by a similar conscious thought, and consequently can have a greater impact on the emotions and behavioral intentions of the dreamer (Morewedge and Norton, 2009). The greater self-insight that people attribute to spontaneous than deliberate thoughts can also result in spontaneous thoughts having a greater impact on the attitudes and perceptions of the thinker (Critcher et al., 2013; Kupor et al., forthcoming). If people believe that spontaneous thoughts reveal as much or more insight into their self and others than do more deliberate and controlled forms of thinking, they may believe that the decisions they make spontaneously reveal their preferences even when the outcome of those decisions are not what they would have chosen if they made that decision deliberately.

The features of a decision may suggest that a spontaneous or intuitive strategy is in some cases preferable to more careful rational deliberation. Rational deliberate strategies are considered superior for choices that are seen as objectively evaluable, sequential, complex, or precise. Sometimes, however, intuitive strategies may be viewed as good or better for choices that are more subjective, holistic, or simple (Inbar et al., 2010). If deliberation leads decision makers to overanalyze a choice and introspect about their preferences to the point of temporarily altering those preferences, deliberation can lead people to choose less satisfying outcomes (Wilson and Schooler, 1991). Participants who thought about the reasons for their preferences prior to choosing one of two posters, for example, reported lower satisfaction with their choice of poster 3 weeks later than did participants who chose one of the two posters without thinking about the reasons for their preferences (Wilson et al., 1993).

In order to directly test mind wandering as a decision strategy, we compared predicted and reported post-choice satisfaction using deliberation or mind wandering as a decision strategy in two experiments. We also compared predicted and reported satisfaction with choice outcomes of both mind wandering and deliberate strategies to predicted and reported satisfaction with random assignment of an outcome. We expected that predicted satisfaction with mind wandering as a choice strategy would be lower than with optimal deliberate choice strategies (Experiment 1), whereas actual post-choice satisfaction with mind wandering as a choice strategy would be as high as with optimal deliberate choice strategies, and higher than random assignment to outcomes (Experiment 2).

## Experiments 1 and 2: deciding by mind wandering

We conducted two experiments comparing predicted and reported post-choice satisfaction between mind wandering as a choice strategy, an optimal deliberate strategy, and random assignment. Specifically, we compared predicted and post-choice satisfaction with a poster chosen from an array of five posters by mind wandering, a deliberate strategy (“choose the best”), and random assignment. We included the random assignment condition as a baseline to allow us to discern if similar ratings in the choice conditions were due to equal satisfaction with the posters selected, to there being no difference in the attractiveness of the posters in the array, or to there being no sensitivity of outcome evaluations to the outcome selection process (i.e., condition assignment).

Predicted and post-choice satisfaction with the chosen alternatives was operationalized as the extent to which participants predicted or reported liking the poster that they received, and the amount of money that participants predicted or reported that they would need to be paid to sell back that poster to the experimenter.

An array of five posters was created from pretest ratings to serve as a consideration set. Forty-three Americans each rated the extent to which they liked or disliked 35 posters (presented in a random order) on an 11-point scale with endpoints, *Dislike Extremely* (1) and *Like Extremely* (11). Color images of each poster were presented alongside the rating scale. Five posters that were similarly attractive formed the consideration set for subsequent studies, all within-subject *t*s_(41)_ ≤ 1.22, all *p*s ≥ 0.23 (range: *M*_most liked_ = 6.16, *SD* = 1.83; *M*_least liked_ = 5.88, *SD* = 1.82).

In Experiment 1, participants made predictions for the post-choice satisfaction of participants who received a poster as a result of mind wandering, an optimal deliberate strategy (choose the best), and by random assignment. We anticipated that forecasters would believe that participants who made a choice by mind wandering would be less satisfied with their poster than those who used an optimal deliberate strategy, but no less satisfied with their poster than participants in the random assignment condition. In contrast, in Experiment 2, we expected that participants who made a choice by mind wandering would be as satisfied with their poster as participants who used an optimal deliberate strategy, and more satisfied with their poster than participants in the random assignment condition.

## Experiment 1: predicted satisfaction

### Materials and methods

#### Participants

One hundred and one residents of the United States of America (33 women; *M*_age_ = 28.98, *SD* = 8.88) completed a survey on the Internet.

#### Procedure

Forecasters were shown the five posters in the consideration set and predicted the extent to which each of three groups of participants in a laboratory experiment would be satisfied with the poster they received. Forecasters were given the exact instructions received by people in each group, were told that the laboratory participants received a full-size print of the poster, and then made their ratings. The random assignment condition was labeled as “Group 1: Now, when you click to continue to the next question, the computer will randomly choose a poster for you from the array of posters.” The mind wandering condition was labeled as “Group 2: Now, please let your mind wander until the poster you feel most drawn to randomly comes to mind. Indicate that poster.” The deliberation condition was labeled as “Group 3: Think carefully about the posters until you identify the poster you most prefer. Indicate that poster.” Choice satisfaction was measured in two ways: Forecasters predicted the extent to which people in each condition would like the poster that they received on a 7-point scales with endpoints, *Extremely Dislike* (1) and *Extremely Like* (7). Forecasters also predicted the smallest amount of money that people in each condition would be willing to accept (on average) to sell their poster back to the experimenter. Predicted willingness to accept was reported on a 15-point analog scale rising in increments of $1 with endpoints, $1 and $15.

### Results

#### Monetary valuation

Predictions for the amount of US dollars that participants were willing to accept to relinquish their poster were submitted to a One-Way repeated ANOVA with three levels of selection process (deliberate choice, mind wandering, random assignment), which revealed a significant effect of condition, *F*_(1, 100)_ = 114.26, *p* < 0.001, η^2^_*p*_ = 0.53. *Post-hoc* tests (Fisher's LSD) confirmed that forecasters believed that people in the deliberate choice condition would require more money to forego their poster than would people in the mind wandering and random assignment conditions, *p*s < 0.001. Forecasters also believed that people in the mind wandering condition would require more money to forego their poster than would people in the random assignment condition, *p* < 0.001 (see Table 1).

**Table 1:** **Predicted valuation and liking for poster received by selection process in experiment 1**.

	**Deliberation**	**Mind wandering**	**Random assignment**
Monetary valuation	$10.99 (3.69)_a_	$9.22 (3.25)_b_	$5.73 (3.37)_c_
Liking	6.26 (1.07)_a_	5.62 (0.97)_b_	3.56 (1.26)_c_

*Means within rows that do not share a common subscript differ by p < 0.05 (Fisher's LSD). Standard deviations are in parentheses*.

#### Liking ratings

Predictions for liking of the selected poster were submitted to a One-Way repeated ANOVA with three levels of selection process (deliberate choice, mind wandering, random assignment), which revealed a significant effect of condition, *F*_(1, 100)_ = 191.75, *p* < 0.001, η^2^_*p*_ = 0.66. *Post-hoc* tests (Fisher's LSD) confirmed that forecasters believed that people in the deliberate choice condition would like their poster more than would people in the mind wandering and random assignment conditions, *p*s < 0.001. Forecasters also believed that people in the mind wandering condition would like their poster more than would people in the random assignment condition, *p* < 0.001 (See Table 1).

### Discussion

Consistent with our predictions, forecasters expected choices made by mind wandering to be less satisfying than choices made by an optimal deliberate strategy. This was true for both ratings of liking and monetary valuations. In Experiment 2, we test our prediction that actual choices made by mind wandering will yield as much satisfaction as optimal deliberation strategies.

## Experiment 2: actual satisfaction

### Materials and methods

#### Participants

One hundred thirty-three Carnegie Mellon University undergraduates (79 women, *M*_age_ = 20.02, *SD* = 1.28) in Pittsburgh, PA received course credit for completing a laboratory experiment.

#### Procedure

Participants saw and inspected images of the five posters (in random positions), after they learned that they would receive a full size poster of one of these five posters in the experiment. Next, participants randomly assigned to the deliberate choice condition were instructed to “Think carefully about the posters until you identify the poster you most prefer” and then indicated that poster in the array. Participants assigned to the mind wandering condition were instructed to “Let your mind wander until the poster you feel most drawn to randomly comes to mind” and then indicated that poster in the array. Participants assigned to the random assignment condition were randomly assigned one poster in the array.

All participants then saw an image of the poster selected and were given a 24″ × 36″ rolled print of it by a research assistant. Next, participants were given the opportunity to sell back their poster to the experimenter. Participants indicated how similar they felt they were to other people receiving their poster on three dimensions: general similarity, conscious thoughts and preferences, and unconscious thoughts and preferences, on 6-point scales with endpoints, *Very Dissimilar* (1) to *Very Similar* (6). The following screen presented participants with an incentive-compatible Becker-DeGroot-Marschak (1964) procedure, consisting of 15 pairs of binding choices: (a) to keep the poster or (b) sell the poster back to the experimenter for a cash sum. The amounts started at $1 and increased in $1 increments to $15. The highest value at which participants chose to keep their poster was recorded as their monetary valuation of the poster. To insure that the task was incentive compatible, participants were informed that at the end of the experiment, one of the values ranging from $1 to $15 would be randomly drawn and they would keep the poster or sell the poster back to the experimenter at that amount, depending on the choice they had made for that pair (e.g., if participants chose, “I would prefer to keep the poster” rather than “I would prefer to sell the poster for the cash payment” at $5 and $5 was chosen, participants would keep the poster). Participants knew they did not have the opportunity to change their mind at a later time.

Participants then reported the extent to which they liked their poster and preferred it to the other four posters in the experiment on 7-point scales with endpoints, *Extremely Dislike* (1) and *Extremely Like* (7), and *Much prefer the other posters* (1) and *Much prefer your poster* (7). After participants provided their demographic information, they were assigned to either keep their poster or sell it back for the cash payment according to their earlier choice.

### Results

One participant was excluded from further analyses because her choices in the BDM procedure were inconsistent and thus impossible to code. The extent to which participants preferred their poster to the other posters and liked their posters was highly correlated, *r*_(130)_ = 0.64, *p* < 0.001 so they were averaged into a single measure of liking.

#### Monetary valuation

We first examined the money participants chose to forgo to keep their poster by condition with a between-subjects ANOVA, which revealed a significant main effect, *F*_(2, 129)_ = 3.34, *p* = 0.04, η^2^_*p*_ = 0.05. *Post-hoc* tests (Fisher's LSD) confirmed that participants in the deliberate choice and mind wandering conditions were willing to forgo larger sums of money to keep their poster than were participants in the random assignment condition, *p*s ≤ 0.04. There was no significant difference in price between the deliberate choice and mind wandering conditions, *p* = 0.88 (see Table 2).

**Table 2:** **Valuation and liking for poster received by selection process in experiment 2**.

	**Deliberation**	**Mind wandering**	**Random assignment**
Monetary valuation	$6.58 (4.38)_a_	$6.71 (4.28)_a_	$4.67 (3.57)_b_
Liking	5.69 (0.86)_a_	5.67 (0.74)_a_	4.01 (1.31)_b_

*Means within rows that do not share a common subscript differ by p < 0.05 (Fisher's LSD). Standard deviations are in parentheses*.

#### Liking

We next examined the extent to which participants liked the poster that they received by condition with a between-subjects ANOVA, which revealed a significant main effect, *F*_(2, 129)_ = 39.12, *p* < 0.001, η^2^_*p*_ = 0.38. *Post-hoc* tests (Fisher's LSD) confirmed that participants in the deliberate choice and mind wandering conditions liked their posters more than did participants in the random assignment condition, *p*s ≤ 0.001. There was no significant difference in liking between participants in the deliberate choice and the mind wandering conditions, *p* = 0.92 (see Table 2).

#### Choice of poster

One obvious possibility is that participants in the deliberate choice and mind wandering conditions exhibited similar valuations and liking for their posters simply because they chose the same posters. Thus, we conducted a χ^2^ Test of Independence to compare the distributions of posters received across conditions. Most important, the distribution of posters received for participants in the deliberate choice and mind wandering conditions differed significantly between conditions, χ^2^_(4, *N* = 90)_ = 9.77, *p* = 0.04. Similarly, the distribution of posters received by participants in the deliberate choice and random assignment conditions differed significantly between conditions, χ^2^_(4, *N* = 87)_ = 15.04, *p* = 0.005. The distribution of posters received by participants in the mind wandering and random assignment conditions, however, only differed marginally in the posters that they received, χ^2^_(4, *N* = 87)_ = 8.38, *p* = 0.08.

Between-subject ANOVAs revealed no significant effects on participants' ratings of similarity to others receiving their poster, so we do not discuss them further, all *Fs* < 1.1, all *p*s > 0.34.

### Discussion

In contrast to the predictions made by participants in Experiment 1, post-choice satisfaction with posters chosen using mind wandering as a choice strategy was as high as with posters chosen by an optimal deliberate strategy. Participants who chose a poster by mind wandering liked and valued their poster as much as did participants who chose the best poster by deliberation, even though the posters they chose were different. Finally, both strategies yielded higher satisfaction than did random assignment to a poster, suggesting that not all posters were equal in desirability and that outcome evaluations were sensitive to the process by which outcomes were selected.

## General discussion

Decisions made by mind wandering appear to be unexpectedly satisfying. People expect to be less satisfied with decisions made by using mind wandering as a choice strategy than with deliberate strategies (Experiment 1). This belief appears, at least in some cases, to be a prediction error. Participants reported similar post-choice satisfaction with decisions made by mind wandering and by deliberation when choosing from consideration sets with real differences (Experiment 2).

The results of Experiment 2 suggested that participants who made a decision by mind wandering did not simply use a deliberate strategy to choose a poster. The posters chosen by participants in the mind wandering and deliberate choice conditions were significantly different. Post-choice satisfaction was lower for participants in Experiment 2 who were randomly assigned a poster than for participants who chose a poster by mind wandering or with an optimal deliberate strategy. The inclusion of the random assignment condition further mitigates concerns that the similar post-choice satisfaction of participants in the mind wandering and deliberate choice conditions was due to participants ignoring the instructions to employ different choice strategies, or resulted from the use of consideration sets with indistinguishably different alternatives. These results do not support the alternative possibilities that participants ignored the instructions or were insensitive to differences in the consideration sets.

The discrepancy between the predicted and actual satisfaction with decisions using mind wandering as a decision strategy may be due in part to detrimental effects of over-deliberation on choice satisfaction (Wilson et al., 1993). Notably, we did not ask participants in the deliberation condition of our study to introspect or list reasons for their preferences (as in Wilson et al., 1993). Instead, they were asked simply to “think carefully about the posters until you identify the poster you most prefer.” As a result, our deliberation instructions likely represent an intermediate level of thought; most importantly, our results extend this previous research by directly comparing predicted and actual satisfaction with decisions made by deliberation to decisions made by mind wandering.

Our findings also contribute to research examining the impact of unconscious vs. conscious decision processes on choice satisfaction (e.g., Dijksterhuis and van Olden, 2006). Unconscious thought has been defined as “object-relevant or task-relevant thought processes that occur while conscious thought is directed elsewhere” and is typically operationalized by having participants complete a distraction task for several minutes, allowing them to continue thinking about the decision only at an unconscious level (Dijksterhuis and Nordgren, 2006, p. 96). The decision strategy of mind wandering could be considered to be a means of instantiating unconscious thought (i.e., people can distract themselves by mind wandering while they continue to direct unconscious thought at the decision problem). Unconscious thought has been argued to perform better than deliberate thought, especially for complex decisions and specifically for the same type of stimuli used in the present studies—art posters (Dijksterhuis and Nordgren, 2006; Dijksterhuis and van Olden, 2006; but see Acker, 2008). Self-paced deliberation has been argued to be an important boundary condition for the superior performance of distraction tasks vs. a constrained time period of forced deliberation (Payne et al., 2008). Because our goal was to compare mind wandering to deliberation, we did not manipulate the amount of time participants were required to spend making their decision in Experiment 2; future research should covary both decision process (mind wandering vs. deliberation) and timing, to examine the joint impact. Our results suggest that people fail to predict that self-paced mind wandering (i.e., engaging in self-distraction) might not leave them less satisfied than self-paced deliberation.

We offer several speculative explanations for the mismatch between predictions and actual outcomes of mind wandering and deliberative decision strategies, and the potential benefits to decision makers. First, deliberation can instantiate post-choice regret. Satisfaction with a chosen alternative is a function of satisfaction with its positive attributes and how it compares with rejected alternatives (Sagi and Friedland, 2007). Unhappiness and regret have been found to be more acute for people who engage in utility maximization strategies than for those who pursue a satisficing strategy (Schwartz et al., 2002). Because mind wandering is associated with worse encoding of external information (Schooler et al., 2011), it may reduce comparison among alternatives and prompt satisficing strategies that reduce feelings of regret relative to more deliberative choice strategies that prompt maximizing strategies. Of course, there are likely decision types in which mind wandering would in fact lead to lower post-choice satisfaction, such as when deciding the guilt of a defendant or whether to undergo a medical procedure (Inbar et al., 2010).

We present evidence from a choice context—an array of art posters—where items were pretested to be minimally different in positive ratings. This consideration set has similar features to other kinds of trivial everyday choices (e.g., toothbrushes, flights, or jams) that have been suggested to make the act of decision making more frustrating or aversive, especially when accompanied by the perception of high decision difficulty or numerous options (Iyengar and Lepper, 2000; Sela and Berger, 2012). We suggest that mind wandering might perform as well as deliberation, in part, because it is less prone to instantiating decision conflict and choice deferral as the result of carefully comparing choice attributes, especially from large consideration sets. Evaluations of cognitive effort expended as the result of mind wandering or deliberation is an interesting question for future investigation. When choosing from a large choice set, mind wandering might be especially likely to leave decision makers feeling less overwhelmed or drained as compared to deliberation. Rather than expending cognitive effort attempting to find a maximizing alternative, people might be just as satisfied forgoing deliberation altogether, and instead letting their mind wander in order to make their choice. Deciding by mind wandering may seem at face value as disturbing as driving on autopilot, but seems to similarly guide one to a satisfactory destination.

### Conflict of interest statement

The authors declare that the research was conducted in the absence of any commercial or financial relationships that could be construed as a potential conflict of interest.
